# Anxiety, coping skills and hypothalamus-pituitary-adrenal (HPA) axis in patients with endometriosis

**DOI:** 10.7243/2054-0841-3-2

**Published:** 2015-06-11

**Authors:** Maria Quiñones, Rebecca Urrutia, Annelyn Torres-Reverón, Katy Vincent, Idhaliz Flores

**Affiliations:** 1 Department of Psychology, Ponce Health Sciences University, Ponce Research Institute, Puerto Rico, USA.; 2 Department of Physiology, Ponce Health Sciences University, Ponce Research Institute, Puerto Rico, USA.; 3 Department of Microbiology, Ponce Health Sciences University, Ponce Research Institute, Puerto Rico, USA.; 4 Department of Biology, Nova Southeastern University, Fort Lauderdale, Florida, USA.; 5 Nuffield Department of Obstetrics and Gynecology, University of Oxford, John Radcliffe Hospital, Oxford, UK.

**Keywords:** Endometriosis, HPA axis, cortisol, anxiety, pain, infertility

## Abstract

**Background:**

Endometriosis is an inflammatory disease that is defined by growth of endometrial tissue outside the uterus, resulting in pain, infertility, and emotional distress. Previous studies have shown that the HPA axis is compromised in patients with chronic, painful diseases, including endometriosis. However, the underlying mechanisms and the physiological and emotional consequences of dysfunctions in the HPA axis in these patients are largely unknown. We aimed to understand whether diurnal circulating cortisol levels in women with endometriosis are affected and how this impacts their emotional and behavioral responses.

**Methods:**

Thirty-two patients with endometriosis and 36 healthy control women provided saliva samples and completed a series of psychological questionnaires. Salivary cortisol levels were measured in duplicate using a colorimetric immunoassay.

**Results:**

There were significant differences in average cortisol levels between endometriosis patients and controls. A negative correlation was found between cortisol levels and infertility and dyspareunia. Furthermore, incapacitating pain was found to be a strong predictor of hypocortisolism. Women with endometriosis reported higher levels of trait anxiety, but showed no differences in perceived stress or in coping styles compared to the control group.

**Conclusions:**

This study supports previous reports of hypocortisolism as a biomarker of aberrant HPA responses in women with endometriosis. Moreover, it provides further insight into the link between HPA axis dysregulation, emotional responses, and the high comorbidity between endometriosis and other inflammatory conditions.

## Introduction

It is well established that inflammatory processes play an important role in the development and pathology of endometriosis [1-3]. Endometriosis is a chronic inflammatory disease of the reproductive system that affects 10% of women during their reproductive years, representing an estimated 176 million patients throughout the world [4-7]. Hypocortisolism has been reported in many chronic disorders including asthma, allergies, major depression, and a variety of chronic pain conditions [8,9]. Specific to pelvic pain, two studies have examined the Hypothalamus-Pituitary-Adrenal (HPA) axis activity in women with endometriosis and one in women with dysmenorrhea Petrelluzzi et al., [10] showed that women with endometriosis had significantly lower salivary cortisol concentrations than controls whilst maintaining normal diurnal variation. Moreover, this study showed that patients reporting higher levels of perceived stress and a poorer quality of life had lower concentrations of salivary cortisol Vincent and colleagues [11]. Showed that in women only suffering with dysmenorrhea (i.e., pain for only a few days a month), serum cortisol levels were significantly lower than in pain-free controls. Additionally, in this study, cortisol levels correlated negatively with the duration of symptoms. On the other hand, Lima et al. have reported that in infertile women with endometriosis serum cortisol levels were higher than in controls [12]. Notably, both high and low cortisol levels are biomarkers of dysfunction of the HPA axis, and they are associated with different psychopathologies [13].

Anxiety, stress, and coping styles have been shown to affect the HPA axis as well as pain perception [14-16]. However, little is known about the coping mechanisms women with endometriosis employ to deal with the physical (pelvic pain) and psychological (stress, anxiety) repercussions of the disease. One study showed that women with endometriosis who suffered persistent post-surgical pain scored higher on a catastrophizing scale [17]. Another found positive correlations between reported coping styles and depression and anxiety levels, and documented associations between pain severity and psychosocial impairments [14]. Pain, especially when chronic in nature, is considered a major cause of physical, psychosocial, and emotional impairment [18,19]. Endometriosis in particular is known to be a source of considerable psychological stress because of its negative effects on not only physical health, but also in the affective and personal life [20]. Emotional distress has been proposed to contribute to the exacerbation of many chronic inflammatory disorders, including endometriosis [21,22]. Women with endometriosis have consistently reported reduced quality of life, high levels of perceived stress and anxiety, and depressive symptoms, which are higher than those reported by patients with other chronic inflammatory disorders [23-25]. In addition, experimental evidence shows that stress exacerbates the size of lesions and inflammatory parameters in a rat model of endometriosis [26], and stress management has beneficial effects [27]. It is possible that a chronic inflammatory response due to dysregulation of the HPA axis is related to the emotional distress and incapacitating pain reported by patients with endometriosis.

The aim of the present study was to examine the relationship between HPA axis functionality, perceived stress and anxiety, and coping styles in a sample of Puerto Rican women with endometriosis. Understanding how these factors contribute to HPA axis dysfunctions will provide further insight into the way women with endometriosis cope with their condition. Our long-term goal is to contribute in the development of tailored strategies for stress and anxiety management, identify potential interventions to ameliorate symptoms, reduce inflammation, and increase the quality of life and productivity of affected women.

## Methods

### Study subjects

Thirty-one women diagnosed with endometriosis (29 via surgery, e.g., laparoscopy; 2 by symptomatology). Thirty-six healthy women without a diagnosis of endometriosis were recruited as controls. Healthy women were recruited through a convenience sample and were within their reproductive age. Patients were recruited through advertisements using the Endometriosis Research Program Patient Registry, and the *‘Fundación Puertorriqueña de Pacientes con Endometriosis’* database and support activities, notice boards, and social media (e.g., Facebook group, Twitter). From those interested in participating, we obtained a saliva sample and data from the study questionnaires, as well as demographic details. The study protocol was approved by the Institutional Review Board of the Ponce Health Sciences University-School of Medicine and (PHSU-PSM). All participants signed an informed consent form prior to participation and were fully informed as to the nature of the study.

### Saliva collection

To measure cortisol levels saliva samples were collected from all participants in sterile tubes and immediately placed on ice. In order to standardize the collection, samples were taken from both patients and controls between the hours of 10:00 am and 2:00 pm, thus controlling for circadian variations in cortisol levels. Participants had been resting for approximately two hours before saliva was collected and were requested to give a sample at least thirty minutes after eating or drinking to prevent contamination. Samples were transferred to the laboratory on ice where they were centrifuged at 2000 rpm for five minutes to obtain a cell-free, clear supernatant. The supernatants were then removed and stored at −20°C for further analysis of cortisol levels.

### Cortisol measurement

Saliva samples were diluted in a 1:2 ratio, and cortisol concentrations were measured using a colorimetric immunoassay, following the manufacturer's protocol (Enzolife Sciences, Farmingdale, NY). Optical densities were measured using a 96-well microplate reader. Cortisol concentrations were then extrapolated using the standard curve. All samples were run in duplicate.

### Surveys and questionnaires

At the time of saliva collection, all participants completed a demographics questionnaire and the Spanish version of the State and Trait Anxiety Inventory (STAI), [28]. Which consists of two scales that measure anxiety as a *state* (i.e., as a response to a specific situation) and as a *trait* (as a general aspect of personality). The State Inventory (STAI-S) is a 20-item scale that assesses anxiety at the time of testing and questions participants about how they feel “at this moment in time” (e.g., the statement “*I feel satisfied*” is rated on a 4-point scale in which 1 signifies “not at all” and 4 denotes “very much”). The Trait Inventory (STAI-T) is a 20-item scale that assesses chronic anxiety. This scale measures how subjects “generally feel” (e.g., the statement *“I take disappointments so keenly that I can't put them out of my mind.”* is rated on a 4-point scale in which 1 signifies “almost never” and 4 signifies “almost always”).

Participants completed additional surveys online. The COPE is a 60-item self-reported questionnaire that evaluates coping thoughts and behaviors in response to stressors [29]. The inventory is divided into three subscales: five scales of four items each measure distinct aspects of *problem-focused* coping; five scales measure aspects considered as *emotion-focused* coping; and three scales measure coping responses in other areas such as focus on and venting of emotions, behavioral disengagement, and mental disengagement. The Holmes and Rahe Stress Scale or Social Readjustment Rating Scale (SRRS) [30] is a 43-item list of stressful events within the past year that could contribute to illness. Patients with endometriosis also completed the Visual Analogue Scale (VAS) to evaluate the severity of their menstrual related pain [31].

### Statistical analysis

Data are presented as means and standard errors. Comparisons were performed using the Mann-Whitney non-parametric t-tests, Fisher's exact test, one-way analysis of variance (ANOVA) and one-way multivariate analysis of variance (MANOVA). The Iterative Grubbs test (alpha=0.05) was used to identify outliers. Spearman correlations between salivary cortisol concentration, infertility (Yes/No), and dyspareunia frequency were calculated. A multiple regression analysis was performed to assess which endometriosis’ symptoms predicted low levels of salivary cortisol. All analyses were conducted using SPSS v17 (IBM, Armonk, New York) and graphs were generated using GraphPad Prism v5.02 (GraphPad Software, La Jolla, CA). Confidence intervals were 95% and p-values less than 0.05 were considered significant.

## Results

### Study population

Study subjects were women of reproductive age, ages 15 to 49 years with surgically diagnosed (n=29) or self-reported endometriosis (n=2) and controls, relatively healthy women who reported not having a diagnosis of endometriosis (n=36). **Table 1** summarizes demographics and clinical characteristics of the study subjects. No significant differences were found between the groups in regards to age, weight, BMI, or age at menarche. Significant differences between groups were seen in the frequency of dyspareunia and infertility (p<0.0001). The most common symptoms reported by women with endometriosis were: back-leg pain (31%), dysmenorrhea (29%), dyspareunia (28%), dyschezia (28%), and incapacitating pain (26%), which are in accord with the literature on endometriosis [32].

### Cortisol levels and correlations with symptoms

**Figure 1:**
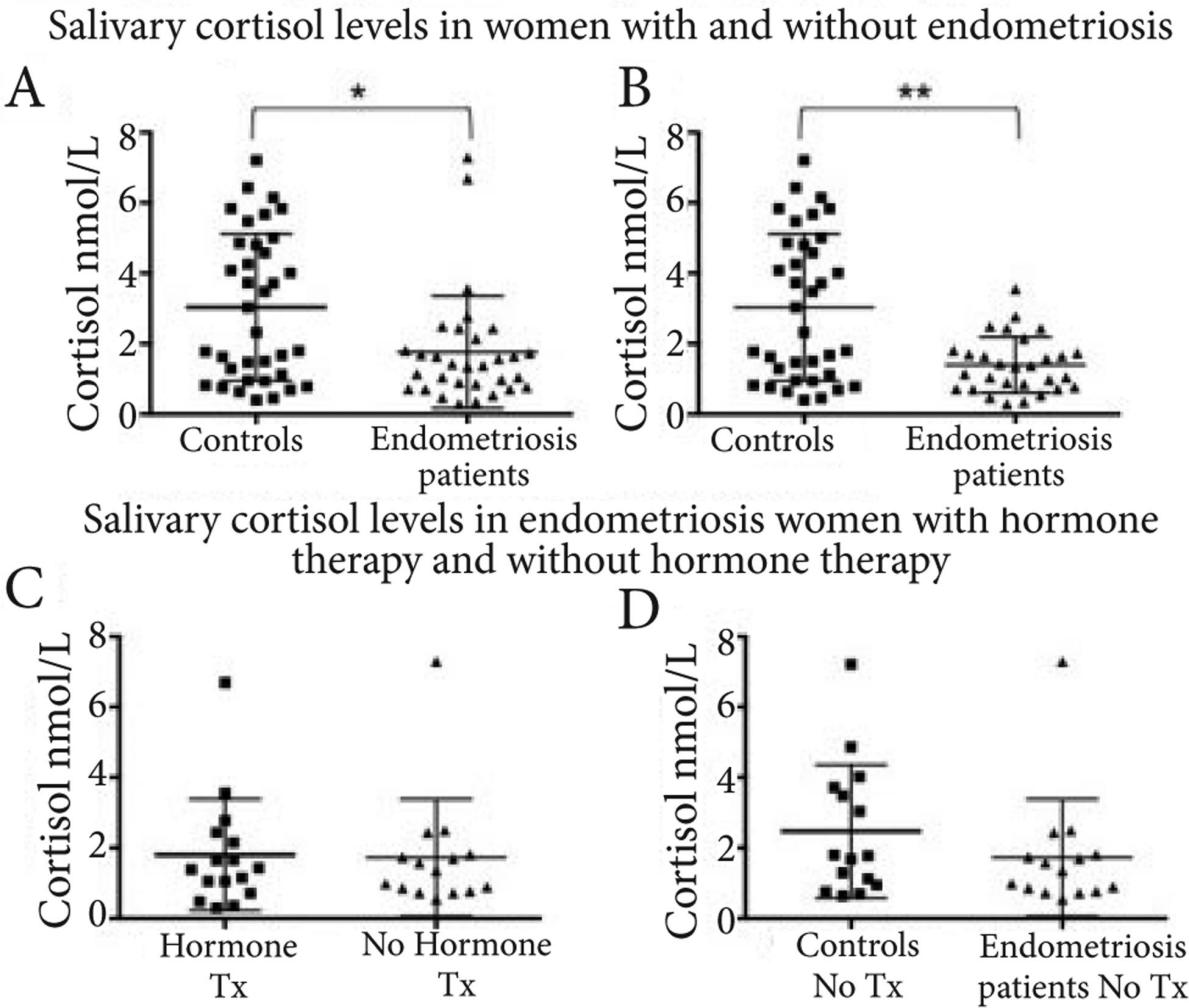
(**A**). Women with endometriosis (n=31) have lower levels of salivary cortisol than health controls (n=36). Bars represent means and standard deviation. (**B**). Using the Iterative Grubbs (alpha=0.05) two outliers were identified in the patients group. Comparisons were done using the Mann-Whitney non-parametric t-test. Statistical significance was set at p<0.05. *p=0.0195; **p=0.0033. (**C**). Cortisol levels were not significantly different in women with endometriosis currently on or off hormone therapy (e.g., oral contraceptives, GnRH agonists, Depo-Provera). Hormone Therapy (Tx) n=16; No hormone Tx n=15. Bars represent means and standard deviation. Comparisons were done using the Mann-Whitney non-parametric t-tests. Outliers not eliminated. Statistical significance was set at p<0.05. *p*=0.9301. (**D**). Cortisol levels had a tendency to be lower in women not currently receiving hormone therapy (e.g., oral contraceptives, GnRH agonists, Depo-Provera) compared to controls. Patients n=15; Controls n=15. Bars represent means and standard deviation. Comparisons were done using the Mann-Whitney non-parametric t-tests. Outliers not eliminated. Statistical significance was set at p<0.05. *p*=0.31.

Chronological age and age at menarche are biological factors known to contribute to diminishing cortisol levels. Therefore, a multiple regression analysis was performed to test the potential confounding of these variables in our study. Since there were no significant differences between age, age at menarche, and cortisol levels in the study sample (*F* (42,2)=0.26, *p*=0.77), we then continued to perform correlations between cortisol and other symptoms. In order to test the hypothesis that women with endometriosis have dysregulation of the HPA axis, we compared mean salivary cortisol levels between patients (1.764 nmol/L; SD=1.56) and controls (3.035 nmol/L; SD=2.087; **Figure 1A**). A Mann-Whitney test indicated that cortisol levels were significantly lower in women with endometriosis than in healthy women (z=2.32; *p*=0.0195). Two patients were excluded from the analysis due to highly abnormal levels of cortisol. When we reanalyzed the data without the patient outliers we found that mean salivary cortisol levels for patients was (1.404 nmol/L; SD=0.795; **Figure 1B**). A Mann-Whitney test indicated that cortisol levels were significantly lower in women with endometriosis than in healthy women (z=2.90; *p*=0.0033).

More women with endometriosis (16 out of 32) reported currently taking hormonal therapies (e.g., oral contraceptives, progestins, GnRH agonists) compared to controls (4 out of 20=20%); however, no significant difference was observed in cortisol levels between women with endometriosis currently taking vs. not taking hormonal therapy (**Figure 1B**). In addition, when comparing only women not currently using hormonal therapy (at least six months prior to sample accrual date), there was still a trend towards lower levels of salivary cortisol in those with endometriosis (n=15) compared to controls (n=15) (p=0.06) (**Figure 1C**). A correlation between anxiety and cortisol levels did not reveal an association in our study (data not shown).

**Figure 2:**
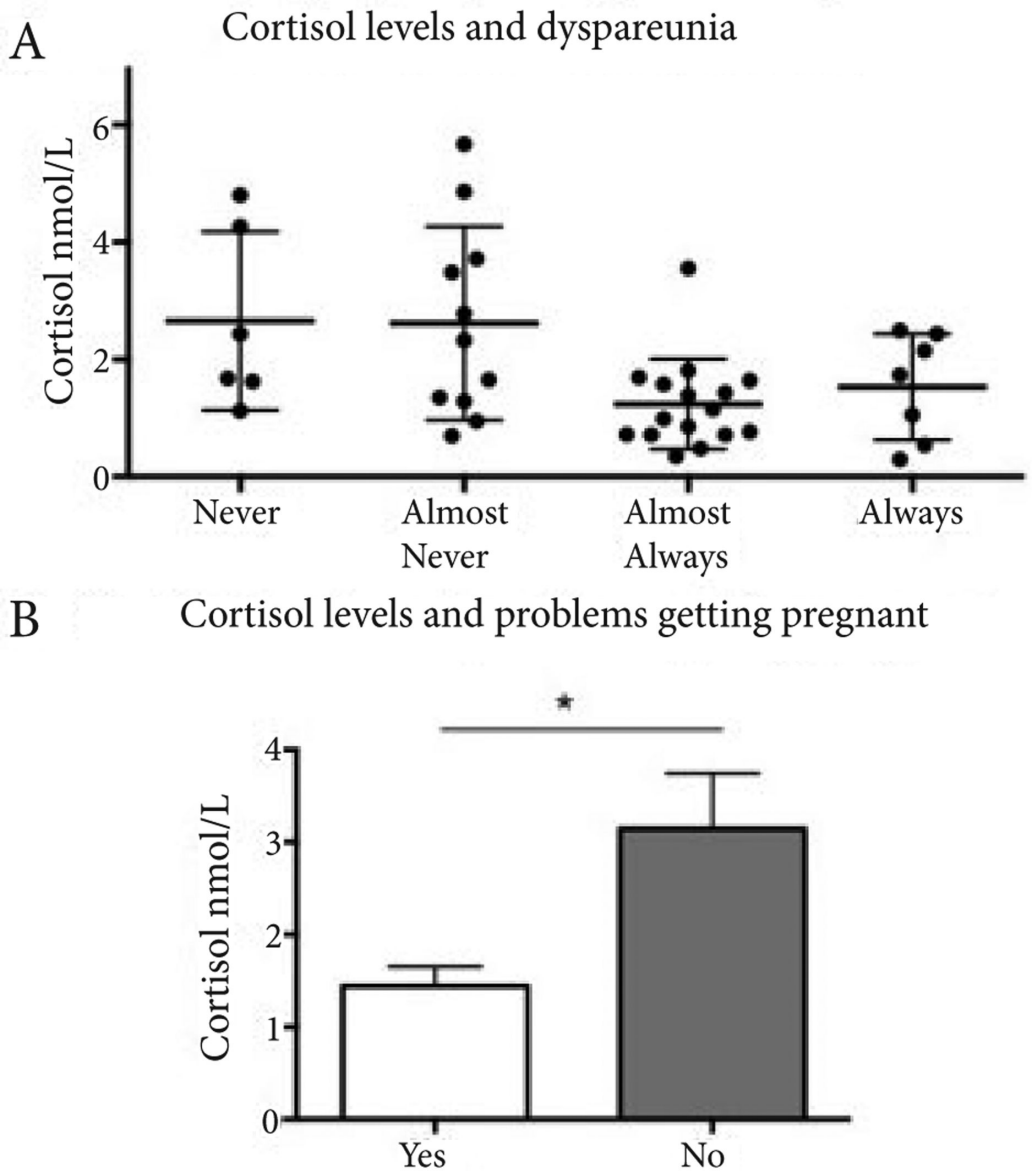
Frequency of dyspareunia (always, almost always, almost never, never) (**A**) and perceived infertility (difficulty getting pregnant, Yes/No) (**B**) are significantly associated with salivary cortisol levels. Statistical differences were determined by Spearman rho correlations for **A**, and by Mann-Whitney non-parametric t-test for **B**. For this analysis the two outliers were excluded. Statistical significance was set at p<0.05.

In order to model the factors that alter HPA axis functioning we next examined the association between cortisol levels and the three most characteristic symptoms of endometriosis: dysmenorrhea, dyspareunia and infertility (defined as difficulty getting pregnant and obstetric history) in all study subjects. Spearman's rho revealed a statistically significant relationship between the frequency with which subjects reported having dyspareunia (always, almost always, never, almost never) and cortisol levels (**Figure 2A**; rs (37)=−0.340, p<0.05). Cortisol levels were lower when women reported frequently enduring severe dyspareunia. Additionally, Spearman's rho revealed a statistically significant correlation between infertility and cortisol levels (**Figure 2B**; rs (22)=0.47, p<0.05). Women who reported experiencing infertility showed lower levels of cortisol as well. Levels of cortisol explained 22.1% of the variance in infertility when reported as a symptom of endometriosis [With infertility: M=1.44, SEM=0.21; Without infertility: M=3.14, SEM=0.61]. Spearman's rho revealed no significant relationship between the frequency with which dysmenorrhea was reported as a symptom and cortisol levels [rs (43)=−0.109, p=0.48].

### Anxiety

**Figure 3:**
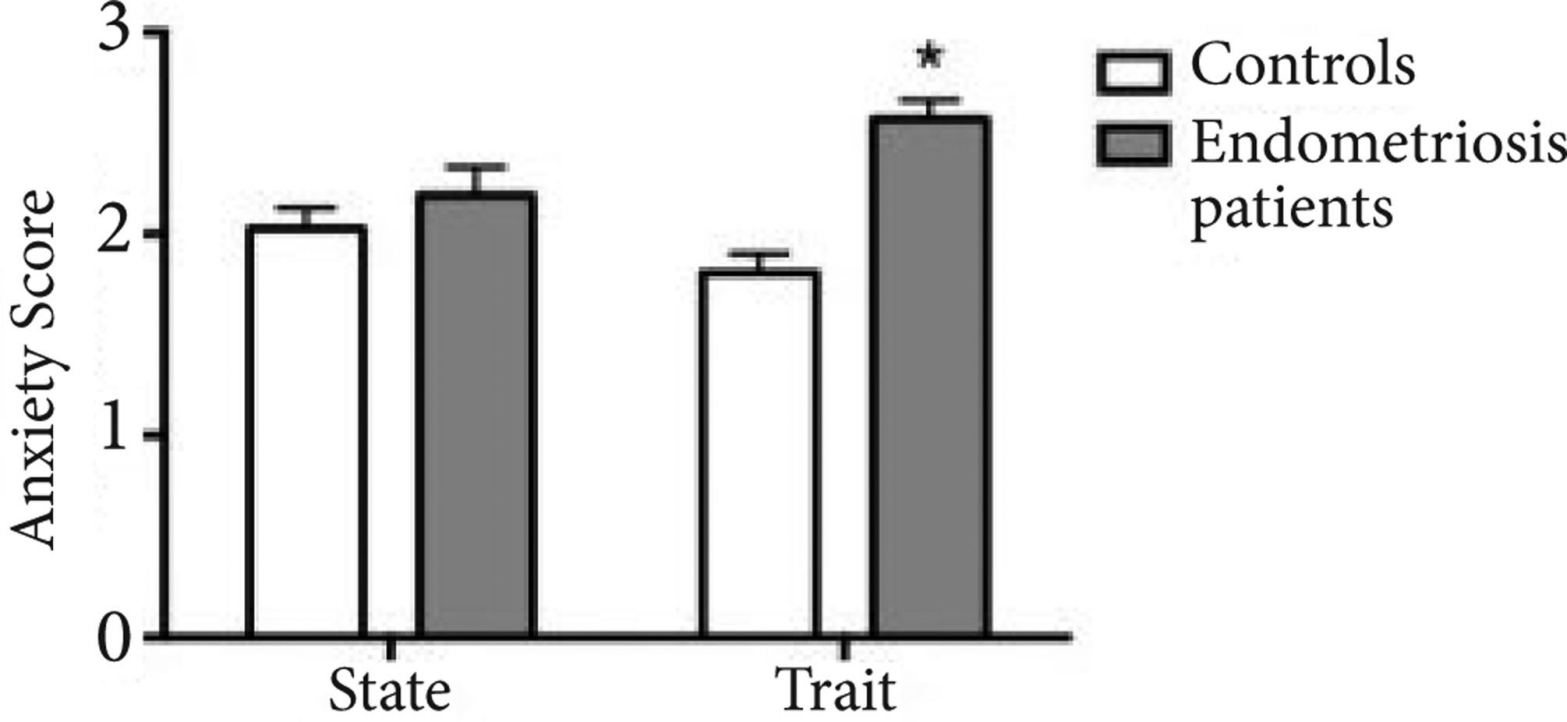
Trait anxiety levels in women with (dark gray) and without (light gray) endometriosis. Women with endometriosis reported a higher score on the anxiety trait subscale (16 out of 29 who completed the survey; 57.1%) compared to controls (3 out of 36; 8.3%). As shown in this figure, a higher proportion of women with endometriosis reported anxiety as a trait. Differences in the trait anxiety subscale scores between groups were significant by Mann-Whitney non-parametric t-test (P<0.01).

We next assessed whether women with endometriosis reported differences in perceived anxiety levels as a *state* (current) or *trait* (persistent or chronic) compared to healthy controls. We observed significant differences between groups with 57.1% (16 out of 29) of women with endometriosis who completed the survey reporting anxiety as a *trait* compared to 8.3% (3 out of 36) of healthy controls (Fisher's exact test, p<0.01) (**Figure 3**). No significant differences were observed in *state* anxiety between the groups.

### Stress

Experiencing stressful events could be expected to influence cortisol levels and may additionally contribute to the experience or exacerbation of symptoms related to endometriosis. Therefore, we evaluated whether women with endometriosis reported experiencing more life changing, stressful events during the last year when compared to healthy controls. A Fisher's exact test revealed no significant differences in the number of stressful events experienced between groups (p=0.262). However, both groups had relatively high stress scores.

### Coping styles

In order to assess if women with endometriosis differ in the way they respond to stress compared to healthy controls, a between subjects MANOVA was conducted. The two dependent variables were problem-focused coping and emotional-focused coping. Analysis of results revealed no significant differences between groups [*F* (2,35)=1.31; *p=0.28*] indicating that both women with endometriosis and healthy women employed these two types of coping strategies equally when responding to a stressful event.

### Predictors of hypocortisolism

A stepwise multiple regression analysis was conducted to evaluate how well characteristic symptoms of endometriosis predicted low levels of cortisol in women with endometriosis. We looked at pain, and somatic symptoms with a total of 11 predictors: dysmenorrhea, dyspareunia, incapacitating pain, muscle pain, pelvic pain, vaginal pain, evacuating pain, urinating pain, profuse bleeding, and irregular bleeding. Since anxiety is known to increase pain perception, anxiety levels were also included in the analysis to control for possible confounding. The model was statistically significant for 1 predictor: incapacitating pain, F (1,14)=6.33, *p*<0.05. This suggests that incapacitating pain was a moderate indicator of low cortisol levels, r=0.56. An ANOVA was conducted to evaluate whether cortisol levels differed by number of symptoms reported by patients with endometriosis. Subjects were divided into two groups based on the number of symptoms reported (1-4 vs. >5) as a way to distinguish between more severe cases, but no significant differences were observed between the groups [*F* (2,1)=0.27, *p*=0.77].

## Discussion and conclusion

The findings of our study add more evidence to the notion that in women with endometriosis HPA responses are impaired as shown by lower basal salivary cortisol levels compared to healthy women. Our results are in agreement with those from studies by [10,11], which also reported hypocortisolism in women with endometriosis and dysmenorrhea, respectively. Low cortisol levels have been reported to have negative side effects on the body's reaction to stress by promoting prolonged inflammatory responses, which may impact the symptomatology of women with endometriosis. Furthermore, dysregulation of the HPA axis has been proposed as a hallmark for chronic pain [33]. Pain is the most characteristic symptom presented by the majority of women with this disease, likely contributing as a significant source of stress. Furthermore, incapacitating pain was the strongest predictor of HPA axis dysfunction in our data set. Our results add to a growing body of evidence reporting a link between chronicity of disease, stress and altered HPA axis activity.

Diverse types of stressors such as physical, emotional, psychological, and social have been shown to activate the HPA axis via different mechanisms [34]. Regardless of the mechanism, dysregulation of the HPA axis by chronic stress leads to long-lasting changes in intracellular signaling pathways that affect the general adaptation syndrome through which the body attempts to minimize harmful effects of stressors. Chronic stress is thought to be associated with activation of cellular immunity, increased production of pro-inflammatory cytokines, release of corticotropin-releasing factor (CRF), acute hyperactivity of the HPA axis followed by a chronic blunted HPA response and glucocorticoid hyposensitivity [35]. Several previous studies have examined both cortisol and CRF in women with endometriosis. Women with mild and moderate endometriosis (stages II and III, American Fertility Society) had higher peritoneal fluid CRF-binding protein levels, suggesting possible changes in circulating levels of CRF [36]. In support of this, CRF levels in ectopic endometrium have been found to be lower during the secretory phase of the cycle as compared to controls [37]. Alterations in corticotrophin releasing factor receptor type 1 (CRFR1) have also been found in endometriotic tissues, which have decreased levels compared to controls through the duration of the menstrual cycle [37]. Similar to our findings, independent studies have found a decrease in salivary cortisol and decreased total follicular cortisol in women with endometriosis [19,38]. However, serum cortisol levels were found to be significantly higher in women with advanced stage endometriosis as compared to controls [12], which might be due to various reasons including differences in patient populations and measurements in serum vs. saliva [39]. Together these studies point towards a desensitization of the HPA axis leading to reduced cortisol secretion. This could result in altered immune responses thus having a negative impact on disease progression and symptoms.

Our data documents significant differences in average salivary cortisol levels between women with endometriosis and controls. Although salivary cortisol levels may not necessarily correlate with cell/tissue effects, this simple test is regarded as a useful biomarker in stress research due to the simplicity of sample collection [40]. Recognizing the complexity in the regulation of the HPA system, we controlled for age, current hormone therapy and time of sample collection (all were collected from 11AM-2PM). Knowing that hormonal therapies influence HPA axis functionality, we compared salivary cortisol levels within the group of women with endometriosis with and without current hormonal treatment and found that both groups showed similar levels, suggesting that hormonal treatment is not responsible for the lower levels of cortisol in endometriosis patients. When we compared only women who reported not currently taking hormonal medications, we could still see a trend (p=0.13) towards lower cortisol levels in women with endometriosis. A larger sample size will be necessary to confirm and validate these results. However, finding women with confirmed endometriosis who are not currently under treatment represented a significant challenge for the current study.

In order to determine if life stressors played a role in the observed levels of cortisol in our study subjects, we asked them to complete the Holmes and Rahe Stress Scale, a survey of past (last year) stressful life events showing no differences in the stress checklist responses between groups and no significant correlation between cortisol levels and reported stressful events. One of the strengths of our study is that we confirmed that the differences in observed cortisol levels were not secondary to a difference in recent stressful events strongly suggesting that the observed hypocortisolism results from a physiological process inherent to the disease. This finding deserves further attention as HPA axis impacts multiple homeostatic processes, from stress perception to immune responses. Future follow up studies should include assessment of current perceived stress levels using available instruments to better understand the relationship between stress levels and HPA activity in endometriosis patients.

In our study, women with endometriosis had higher levels of *trait*, but not *state*, anxiety. *Trait* anxiety differs from *state* anxiety in the intensity, duration and range of situations in which the person experiences anxiety. Therefore, while anxiety as a state is considered a transient and common emotional reaction, anxiety as a trait may represent a general disposition towards constantly experiencing anxiety [41]. Endometriosis appears to be a predisposing factor to suffer from trait anxiety, which we propose to be due to the chronicity of the pain and high anticipatory stress. In support of this, a positive correlation between pain intensity and trait anxiety was previously observed in patients with endometriosis [20]. Anxiety as a trait has also been reported to be high in various disease states and positively correlated with pain scores. Visceral pain sensitivity is positively associated with serum cortisol levels, which may relate to the immunosuppressive effects of cortisol, while high levels of state anxiety predicted a lower pain threshold [42]. Thus, while *state* anxiety leads to higher cortisol, *trait* anxiety is negatively associated with cortisol levels [43], strongly supported by our results.

Since individuals use different coping strategies depending on the stressful context, we investigated whether women with endometriosis engaged in different coping styles compared to healthy controls. The results on this study yielded no significant differences in the coping styles used by women in both groups. Also, there were no correlations between coping styles and cortisol levels. Our findings contradict previous studies with chronic diseases where patients tend to report using more avoidant, passive or emotional coping strategies [44,45]. Taken together, these data support the notion that hypocortisolism is due to inherent factors in women that may predispose them to endometriosis and other inflammatory conditions secondary to the experience of chronic pain.

It could be speculated that endometriosis symptoms may have chronic, persistent impact in the emotional well being of the patients, resulting in aberrant HPA responses and hypocortisolism. In support of this hypothesis, we observed significant correlations between cortisol levels and dyspareunia and infertility (but, interestingly, not to dysmenorrhea). It is also possible that aberrant HPA responses are associated with the underlying etiology of these two characteristic symptoms of endometriosis. While dysmenorrhea affects a wide range of women (i.e., is common but not a specific symptom of endometriosis) and is present in various gynecologic disorders, dyspareunia and infertility are commonly seen occurring together in women with endometriosis. Also, cortisol levels were not associated with years with symptoms, which is not in accord with observations of [11]. This discrepancy might be partially explained by different ethnic groups, cultural differences in pain and stress perception, and the current study limitations of a small sample size. Multiple regression analysis showed no significant correlation between any of the symptoms reported and cortisol levels, nor between those patients reporting more than four symptoms (representing a more severe disease phenotype) compared with women reporting fewer symptoms. Importantly, low levels of cortisol were primarily predicted by incapacitating pain. Incapacitating pain has been regarded as a highly predictive sign of endometriosis [46]. Furthermore, incapacitating pain can also be considered a substantial source of anticipatory anxiety if chronically present. This provides further support for our findings regarding trait anxiety, which may result from the anticipation that they will have to endure severe pelvic pain once again.

Whether hypocortisolism predisposes to endometriosis, or vice versa, is still unknown. It is possible that painful symptoms act as stressors to suppress cortisol, which then alters the immune environment and leads to disease exacerbation and increased symptomatology. This is a logical hypothesis due to the high comorbidity between endometriosis and other inflammatory conditions such as asthma, allergies, and irritable bowel syndrome [47,48]. Hypocortisolism may result in a hyperactive immune system, a lowered resistance to disease, and a tendency for a prolonged inflammatory response. This hypothesis would also agree with our finding that there was no correlation between cortisol concentration and years with symptoms. The patients in the study had been experiencing symptoms for varying amounts of time, ranging from 12 months to 18 years. Thus, the hypocortisolism observed might imply that in women with endometriosis, even a short (e.g., 1 year) exposure to pain might be sufficient to negatively impact the HPA axis. Further studies are needed to verify the direction of this relationship and to determine the cause of hypocortisolism in women with endometriosis, for which the animal model might prove very useful.

Because endometriosis also has very limited treatment options, further studies could examine the efficacy of hormone-based therapy to regulate cortisol levels. In theory, regulating cortisol levels would help suppress the immune response and control inflammation, possibly having an effect on the development or manifestation of symptoms. Complementary therapy alternatives such as physical therapy, stress management, and psychological interventions have proven useful for the management of chronic inflammatory disorders [49-51]. It still needs to be determined how these interventions directly impact HPA axis activity, thus additional experimentation is required to find treatment alternatives for this debilitating disease. Finally, our observation that incapacitating pain is the strongest predictor of hypocortisolism, and given the negative consequences of low cortisol levels documented here and by other studies, provide strong support for prompt and adequate treatment of pain in women with endometriosis.

## Figures and Tables

**Table 1:** Demographics of patients with endometriosis relative to control group.

	Patients with endometriosis (n=29)	Control group (n=29)
Age	29.1 (1.2)	31.7 (2.4)
**Menstrual cycle**		
Regular	9 (31.0%)	15 (51.7%)
Irregular	20 (60.9%)	14 (48.3%)
BMI (kg/cm^2^)	25 (1.2)	25.1 (0.9)
**Relationship status**		
Single	12 (41.4%)	19 (65.5%)
Married	13 (44.8%)	10 (34.5%)
Consensual union	2 (6.9%)	--
Divorced	2 (6.9%)	--
**Education**		
1 to 12 years	3 (10.3%)	--
Some college	9 (31%)	6 (20.7%)
Bachelor's degree	7 (24.1%)	9 (31%)
Master's courses	1 (3.4%)	2 (6.9%)
Master's degree	8 (27.6%)	2 (6.9%)
Doctoral courses	--	6 (20.7%)
Doctoral degree	1 (3.4%)	3 (10.3%)
Medical courses	--	1 (3.4%)

## List of abbreviations

CRF: Corticotropin releasing factor
HPA: Hypothalamus pituitary axis
GnRH agonists: Gonadotropin releasing hormone agonists
VAS: Visual analogue scale
STAI: State and trait anxiety inventory
SRRS: Social readjustment rating scale
